# Why we need families in genomic research on developmental psychopathology

**DOI:** 10.1002/jcv2.12138

**Published:** 2023-01-27

**Authors:** Rosa Cheesman, Ziada Ayorech, Espen M. Eilertsen, Eivind Ystrom

**Affiliations:** ^1^ PROMENTA Research Center Department of Psychology University of Oslo Oslo Norway; ^2^ Centre for Fertility and Health Norwegian Institute of Public Health Oslo Norway; ^3^ Department of Mental Disorders Norwegian Institute of Public Health Oslo Norway

**Keywords:** genomics, psychopathology, twin and family studies

## Abstract

**Background:**

Fundamental questions about the roles of genes, environments, and their interplay in developmental psychopathology have traditionally been the domain of twin and family studies. More recently, the rapidly growing availability of large genomic datasets, composed of unrelated individuals, has generated novel insights. However, there are major stumbling blocks. Only a small fraction of the total genetic influence on childhood psychopathology estimated from family data is captured with measured DNA. Moreover, genetic influence identified using DNA is often confounded with indirect genetic effects of relatives, population stratification and assortative mating.

**Methods:**

The goal of this paper is to review how combining DNA‐based genomic research with family‐based quantitative genetics helps to address key issues in genomics and push knowledge further.

**Results:**

We focus on three approaches to obtaining more accurate and novel genomic findings on the developmental aetiology of psychopathology: (a) using knowledge from twin and family studies, (b) triangulating with twin and family studies, and (c) integrating data and methods with twin and family studies.

**Conclusion:**

We support the movement towards family‐based genomic research, and show that developmental psychologists are particularly well‐placed to contribute hypotheses, analysis tools, and data.

## Graphical abstract

There are major stumbling blocks in the genomics of psychopathology. This review shows how more accurate and novel genomic findings on the developmental aetiology of psychopathology can be obtained by (a) using knowledge from twin and family studies, (b) triangulating with twin and family studies, and (c) integrating data and methods with twin and family studies.

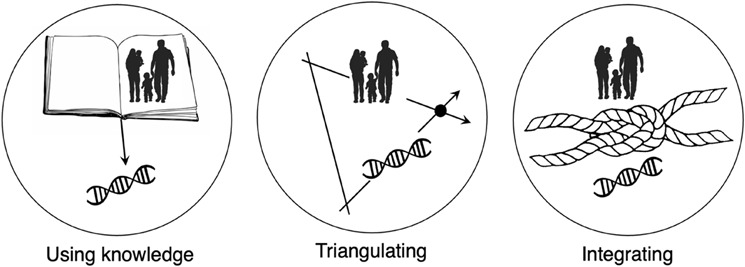

**Key points**

- Population‐based unrelated samples have provided exciting new insights into the genomics of psychopathology, but key challenges remain, including missing heritability and difficulty distinguishing genetic, environmental, and confounding influences.
- We outline three ways in which family information can be used to combat these issues in genomics: (a) using knowledge from twin and family studies even in the absence of family data (b) triangulating population‐based genomic approaches against twin and family methods, and (c) fully integrating genomic data and methods with twin and family studies.
- Developmental psychologists with expertise in family research are well‐placed to capitalise on these strategies to accelerate genomic discoveries on childhood psychopathology.

## INTRODUCTION

Psychiatric disorders such as substance use, depression and anxiety, and schizophrenia run in families (Steinhausen et al., 2009). In trying to disentangle the effects of genes and environment, this observed resemblance between relatives has been of central concern. Family‐based quantitative genetic studies, involving not only classical adoption and monozygotic (MZ)‐dizygotic (DZ) twin designs but also children of twins and in vitro fertilisation designs (Eaves et al., 1978; Pingault et al., 2018), have demonstrated replicable patterns of heritable and environmental influences (Plomin et al., 2016). Both genetic and environmental factors are always important, and more complex extended models have shown how genetic and environmental influences play out over time and depend on one another. For example, genetic factors are the largest contributor to continuity in emotional symptoms across ages, while environmental factors tend to explain symptom changes (Waszczuk et al., 2014). Parents' heritable traits play a role in how positively they treat their adolescent offspring (Marceau et al., 2016) and children's heritable traits may evoke controlling parenting (Eley et al., 2010).

With the characterisation of the human genome and development of cheaper genotyping technologies, the zeitgeist turned towards scanning for specific genetic variants (single nucleotide polymorphisms (SNPs)) associated with psychopathology. The essence of this genome‐wide association study (GWAS) paradigm was based on quantitative genetics, with the focus on many genetic effects (polygenicity), and the implementation of GWAS as a linear regression of a trait on SNP dosage (0, 1, or 2 copies of an allele) (Visscher & Goddard, 2019). This convergence of quantitative and molecular genetics paved the way to modern behavioural genomics (Plomin, 2022). However, genomic research initially left behind family‐based samples and methods. Population‐based samples excluded relatives to control for confounding from common environmental influences. Genomic methods were developed that could estimate heritability from SNPs without requiring specific family structures. For the first time, individuals could be given measures of their genetic risk for psychiatric disorders (i.e., polygenic scores (PGS), also known as polygenic indices). However, there are many challenges at the cutting edge of genomic research in developmental psychopathology. Here, we review two (related) challenges of utmost relevance to this field, leaving others for more general articles (Brandes et al., 2022).

### Challenge 1: Missing heritability

**FIGURE 1:**
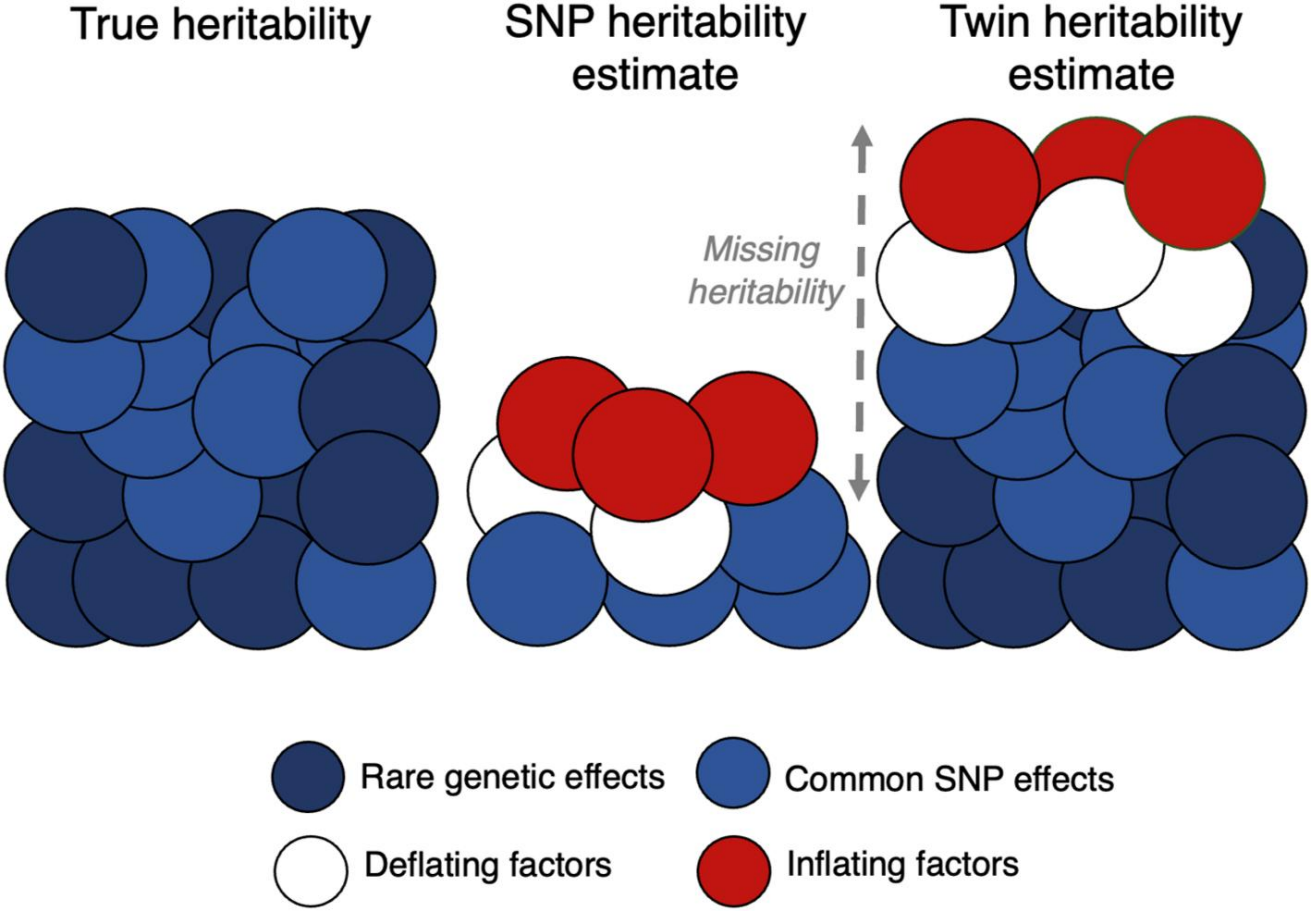
Estimating heritability. Heritability is the proportion of phenotypic variance among individuals that can be attributed to genetic differences in a certain population. Figure 1 sketches the heritability of an imagined phenotype in an imagined population (left, composed of direct effects of common and rare genetic variants), and how accurately population‐based SNP heritability estimates (middle) and classical MZ‐DZ twin heritability estimates (right) are likely to capture this under certain circumstances. SNP heritability estimates include only genetic effects tagged by SNPs included in the analysis, whilst twin heritability estimates capture effects of all DNA differences in the sample. Various factors can lead heritability estimates to be overestimated (red circles increasing the size of the estimate) or underestimated (white circles representing empty space reducing the size of the estimate). The impact of each biasing factor is population and phenotype‐dependent (the number of red vs. white circles would vary across phenotypes), and factors do not necessarily work in the same way for SNP‐ and twin‐based methods (some factors are red for SNP heritability but white for twin heritability e.g., assortative mating). SNP heritability estimates can be inflated by indirect genetic effects, assortative mating, and population stratification (red), but deflated if genetic effects covary negatively with indirect genetic effects (white). Twin heritability estimates can be inflated if the equal environments assumption does not hold (i.e., greater environmental sharing among MZ than DZ twins) and in the presence of non‐additive genetic effects and gene‐by‐shared environment interaction and deflated in the presence of assortative mating. Missing heritability (dashed grey arrow) is the gap between SNP‐and twin‐based heritability estimates. A key explanation for missing heritability is that SNP heritability estimates cannot capture rare genetic effects. Deflating and inflating factors make it difficult to assess missing heritability. For example, the missing heritability problem could be more severe than assumed if assortative mating is inflating SNP heritability estimates and deflating twin heritability estimates.

While heritability estimates from classical twin studies involve assumptions (e.g., random mating and equal environmental sharing for MZ and DZ twin pairs; see Figure 1), they provide a useful benchmark for the total effect of all kinds of DNA differences in the population. In the modern DNA‐era, it has proven difficult to capture the full magnitude of twin‐based genetic influence on complex traits with directly measured SNPs—known as the ‘missing heritability’ problem (Maher, 2008; Manolio et al., 2009). SNP heritability is by definition smaller than twin heritability, since only effects tagged by measured or imputed common variants are captured, and not rare variants or de novo mutations (Yang et al., 2017). However, for childhood psychopathology in particular, the missing heritability problem has been particularly severe. Across 37 measures of childhood psychopathology in the UK Twins Early Development Study, the average SNP and twin heritabilities diverged widely at 6% and 52%, respectively (Cheesman et al., 2017). Moreover, recent GWAS of general psychopathology and internalising problems found low SNP heritabilities of 5% and 2%, respectively (Jami et al., 2022; Neumann et al., 2020). If SNPs truly explain this little variance in childhood psychopathology, this casts doubt on the value of conducting and interpreting genomic research using methods that are limited to additive effects of common variants. Later, we explain how family data can shed light on key contributors and biases (see Figure 1) and recover missing heritability, thus increasing the utility of genomic research on psychopathology.

### Challenge 2: Understanding genetic, environmental, and confounding effects

**FIGURE 2:**
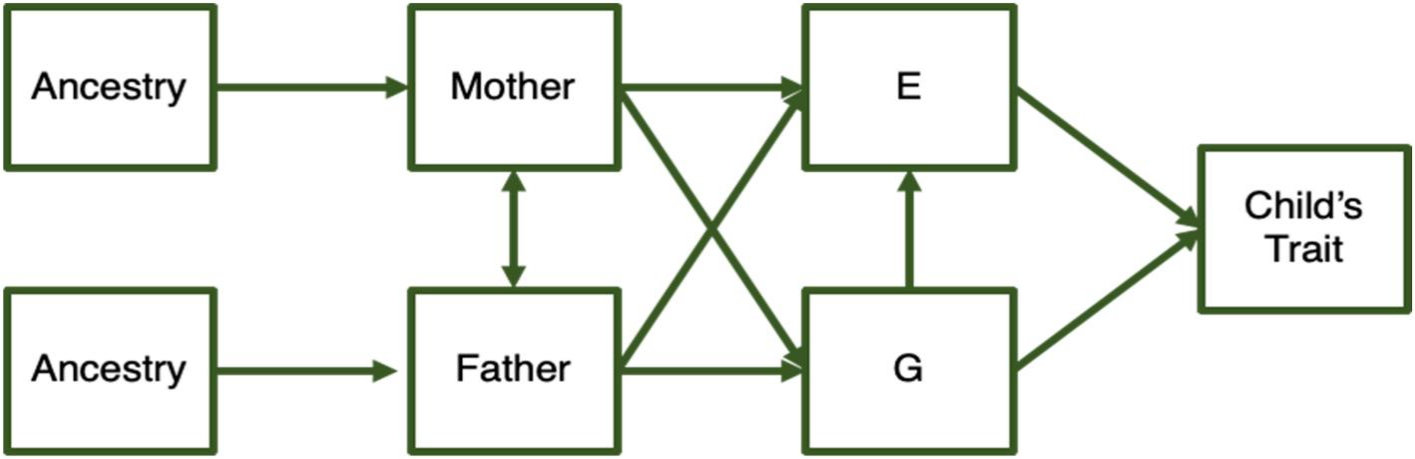
Genetic, environmental and confounding influences on childhood psychopathology. Note that G and E represent the genetic and environmental influences on a child's trait. A **direct genetic effect** is the effect of the child's genetic variation on their own trait, through the pathway: Mother/Father –> G –> Child's trait. As well as transmitting genes, parents provide part of the child's environment. These genetic and environmental influences are not independent, because the environmental effect on the child trait may be partially influenced by parental genotype. Indeed, an **indirect parental genetic effect** is an environmentally‐mediated effect of the parental genome on the offspring phenotype, such as when parental genetic risk for depression impacts on child depression via parental emotional symptoms (Cheesman, Eilertsen, et al., 2020). Indirect parental genetic effects are included in the diagram through the following pathways: Mother/Father –> E –> Child's trait. Note that whilst PGS for non‐transmitted alleles can be used to capture indirect parental genetic effects (Kong et al., 2018), they are not the same thing, since the former can also arise from transmitted parental alleles. The path from G to E represents **evocative and active gene‐environment correlation**, and the presence of paths from Mother/Father to both G and E reflect **passive gene‐environment correlation**. **Assortative mating** occurs when there is greater similarity between partners than expected by chance and is reflected by the double headed arrows running between mother and father. When mating is influenced by heritable characteristics, this results in increased trait‐specific genetic and phenotypic variance in the child generation. **Population stratification** can be described as confounding due to ancestry differences in the population. Genetic differences between subpopulations (with different allele frequencies) can become correlated with phenotypic differences (in parents and/or children) even if they do not have a causal effect on the trait.

A causal direct genetic effect means that a genetic substitution would lead to a change in an individual's own phenotype (albeit a small effect for a single SNP, since complex human traits are highly polygenic). Conventional genomic studies based on samples of unrelated individuals are unable to distinguish direct genetic effects on psychopathology from indirect genetic effects, assortative mating, and population stratification (Barry et al., 2022; Morris et al., 2020; Young et al., 2019) (see Figure 2 for definitions). Although these factors threaten interpretation of genetic associations in many kinds of studies, they are also of interest in themselves. Importantly, studies of parental indirect genetic effects provide a new way to quantify the impact of parental behaviour on child psychopathology. Indirect parental genetic effects measured with SNP‐based methods appear to be important contributors to variation in developmental psychopathology, explaining ∼15% of the variance in Attention Deficit/Hyperactivity Disorder (ADHD) and depression symptoms (Cheesman, Eilertsen, et al., 2020; Eilertsen et al., 2022). Later, we review how genomic data on families can help dissect genetic associations with psychopathology into direct and indirect genetic effects, and in turn parse indirect genetic effects from bias due to assortative mating and population stratification. We call the section ‘understanding’ these effects because we are now using family data in various ways not only to quantify them, but to uncover how they work for example, environmental mediation of direct and indirect genetic effects across development. Quantifying is necessary before understanding mechanisms.

### The current review

**FIGURE 3:**
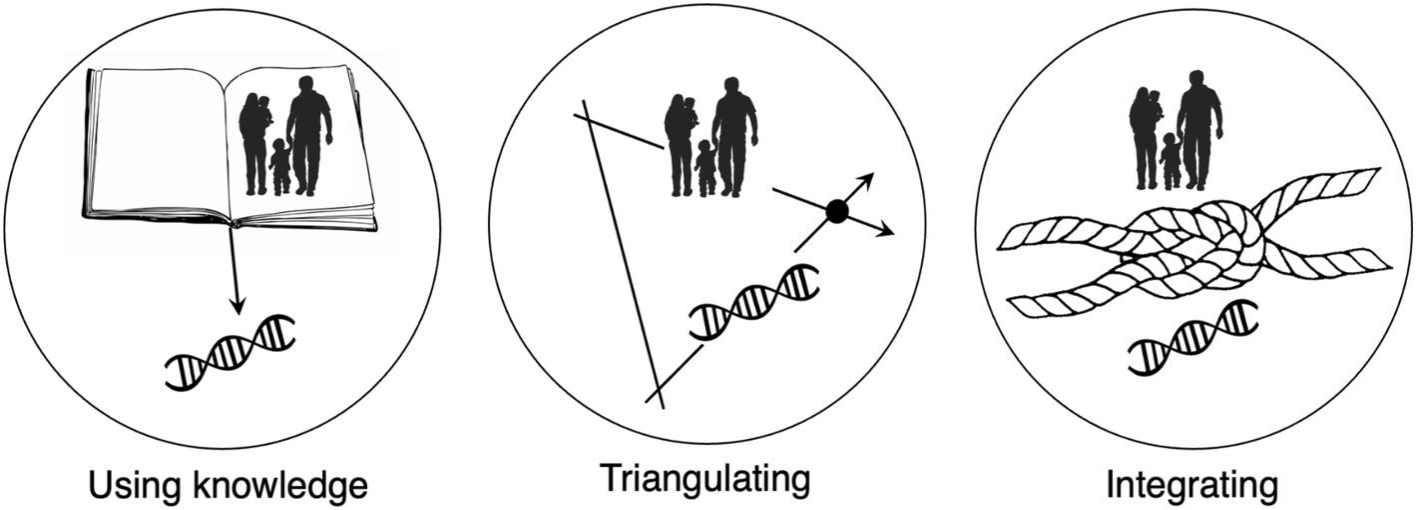
Leveraging family data in genomics in three ways. The present article puts forward three ways in which family data can be used to advance knowledge on the genomics of childhood psychopathology: using knowledge, triangulating, and integrating. For example, knowledge from twin research on gene‐environment correlation can help us to interpret population‐based genomic associations with psychopathology and generate testable hypotheses about mediating mechanisms. Triangulating twin and genomic methods with different assumptions in the same dataset can, for example, allow us to quantify the role of parental behaviour relative to other environmental influences on psychopathology. Integrating family and genomic data can help to recover missing heritability by capturing rare genetic effects that are missed in population‐based studies of unrelated people.

As shown in Figure 3, this review presents three ways in which twin and family information are vital for overcoming key challenges in genomics: the missing heritability problem (challenge 1), and distinguishing between genetic, environmental, and confounding effects (challenge 2). We focus on uses of various genetic methods, rather than detailing the nuts and bolts of how methods work (see (Barry et al., 2022; McAdams et al., 2022)).

## USING EXISTING KNOWLEDGE

The developmental behavioural genetics literature provides a wealth of knowledge on genetic architecture and gene‐environment interplay across development. This knowledge, rooted in family‐based research, can fuel advances in genomic research on psychopathology, even when genomic data on families is unavailable.

### Using existing knowledge to address missing heritability

Genomic study results depend on accurate phenotypic measurement. Twin studies have a long history of combining psychometric modelling with heritability estimation. This literature has informed work tackling the low SNP heritability of childhood anxiety and depression. Such emotional problems are typically transient and show relatively low time‐specific genetic influence. However, any stability in symptoms across time is more strongly influenced by stable genetic influences (Waszczuk et al., 2016). A measure capturing stability in emotional symptoms over time (i.e., common variance in symptoms across ages 7, 12 and 16) showed a higher SNP heritability than for time‐specific measures (15% vs. 5% on average, respectively) (Cheesman et al., 2018). This twin study‐inspired approach of leveraging longitudinal data improved power for polygenic scoring and genetic correlation analyses. Although this does not narrow the gap between twin and SNP‐heritability (because both are higher for the stable internalising measure), anything that increases SNP‐heritability from a negligible level is useful. Going forward, GWAS of internalising should focus on a more heritable, temporally stable phenotype to aid gene discovery.

Similarly, twin studies have often adopted a multivariate approach looking beyond typical boundaries between traits and diagnoses. Given that phenotypic covariance between traits is substantially caused by genetic covariance (Plomin et al., 2016), multivariate approaches are useful for maximising SNP heritability. The replicated twin‐based evidence for strong genetic overlap between ‘externalising’ phenotypes such as ADHD, alcohol dependence, smoking, and risk‐taking behaviour (Achenbach, 1966; Hicks et al., 2013), has been leveraged for genetic association analysis using Genomic Structural Equation Modelling (Karlsson Linnér et al., 2021). Here, statistical power was increased by pooling data across genetically correlated traits and assessing SNP effects on overall externalising liability. The added value of multivariate genetic research methods in the genomic era is that the externalising phenotypes do not all need to be measured within the same individuals. Biological insights that generalise across diagnoses could be useful for developing widely applicable treatments.

As mentioned above, approaches to refining phenotypic measurement raise SNP heritability but do not narrow the gap with twin heritability estimates. In future, knowledge from twin and family studies could be used to close the missing heritability gap. For example, existing evidence suggests that any non‐additive genetic effects and gene‐by‐shared environment interactions will be captured in estimates of twin heritability. In contrast, these effects are not part of SNP heritability estimates, which only consider additive genetic effects and do not compare individuals who share a family environment. Since it is much easier to explore non‐additive genetic effects and gene‐by‐shared environment interactions with twin/family data, the relevance of these factors for specific psychopathology phenotypes in the twin literature should be reviewed before pursuing genomic approaches, which require vast sample sizes (Yang et al., 2017). For example, given the role of non‐additive genetic effects in childhood autism traits (Cheesman et al., 2017), dominance‐genome‐wide association study methods (Okbay et al., 2022) could be particularly fruitful.

### Using existing knowledge to understand genetic, environmental, and confounding effects

Recently, the quantification of direct genetic effects and ‘genetic nurture’ (parental indirect genetic effects) using family‐based genomic methods is leading to new insights on psychological phenotypes (see ‘Integrating designs to estimate genetic, environmental, and confounding effects’). Somewhat overlooked in this newer literature is the extensive theoretical and empirical knowledge on how parenting is influenced by heritable characteristics (Plomin & Bergeman, 1991; Plomin et al., 2016). Figure 2 visualises the well‐established gene‐environment correlation mechanisms, including how children passively inherit genetic variants that are also present in parents and influence how the family environment is shaped (passive gene‐environment correlation). Data on extended twin families have been used for decades to estimate parental indirect genetic effects on offspring phenotypes (Jami et al., 2020; Magnus, 1984). Given the existing knowledge from family and twin studies, it is unsurprising that conventional population‐level genomic associations capture environmental effects from parents (passive gene‐environment correlation) (Friedman et al., 2021; Ystrom, 2019).

Although we can now estimate direct and indirect genetic effects using genomics methods, this does not mean that we can explain them. The family‐based literature also helps us to interpret our results with a wider range of explanatory mechanisms in mind. Complex human phenotypes are not simply ‘in‐born’. Genetic influences play out through gene‐environment correlation mechanisms involving the active selection of environments and evoked reactions from others. A replicated example of a technically ‘direct’ genetic effect that is not purely biological as it sounds is that SNPs become associated with lung cancer because they predispose individuals to heavy smoking (Gage et al., 2016). Similarly, staying in education until doctoral level does not simply happen due to the possession of a set of genetic variants. Direct genetic effects are mediated through reinforcement from parents and teachers, and the active pursuit of educational environments such as more advanced mathematics classes (Harden et al., 2020). Indirect genetic effects could also include wide‐ranging and distal environmentally mediated pathways which have not yet been explored, such as parental genetic risk for psychiatric disorders influencing movement to urban neighbourhoods, which in turn may influence their children's traits. Nonetheless, separating direct and indirect genetic effects is useful for distinguishing groups of mechanisms. For instance, children's ADHD polygenic score correlates independently with household chaos after controlling for mothers' ADHD polygenic score, suggesting that children contribute to household chaos through evocative or active gene‐environment correlation, rather than passive gene‐environment correlation (that is, the child's score tagging indirect effects of parental ADHD genetic risk) (Agnew‐Blais et al., 2022).

Acknowledging the complexity of gene‐environment correlations and dynamic mutual influences of family members over time (Ahmadzadeh et al., 2019; Tucker‐Drob & Harden, 2012), which is background knowledge from twin and family studies, is essential to interpreting genomic studies. This body of work helps us to move away from a static view of aetiology. We could apply techniques from longitudinal twin studies to study unanswered questions in genomics such as whether the same heritable traits contribute to parental indirect genetic effects across forms of psychopathology, and across childhood and adolescence. This knowledge could reveal improved interventions targeted to certain symptoms and developmental windows.

## TRIANGULATING DESIGNS

Methods for estimating genetic and environmental influences are imperfect in different ways. If we know what the differences are, then directly comparing results across designs can help to quantify biases, and patch together a more realistic picture than possible with a single design. Triangulation refers to this strategic use of multiple methods to address one question (Munafò et al., 2021). Here, we focus on the benefits of triangulating family‐based designs with genomic designs, as well as triangulating integrated within‐family genomic designs.

### Triangulating designs to address missing heritability

Triangulation between the classical twin design and new genomic heritability methods allowed the missing heritability problem to be discovered and quantified. Although these are both heritability methods, they rely on different assumptions and data. Existing results from comparing twin and SNP‐based estimates suggest that common SNPs hardly contribute any variance in childhood psychopathology. Particularly useful insights on the causes of low SNP heritability come from comparing results from different methods applied to the same sample. For example, low SNP heritabilities in large GWAS meta‐analyses are often pinned on heterogeneity of measurement between cohorts within GWAS, as well as differences between GWAS and twin phenotyping and ascertainment. However, SNP heritabilities for individual psychopathology measures are much lower than twin estimates even within a single large homogeneous sample (Cheesman et al., 2017), suggesting that heterogeneity is not necessarily the key factor for childhood psychopathology.

### Triangulating designs to understand genetic, environmental, and confounding effects

To accurately estimate how important parents are in the development of childhood psychopathology, we can compare twin‐based shared environmental variance components and SNP‐based parental indirect genetic effects estimated in the same sample. Shared environmental variance does not only capture family environmental effects, but anything non‐genetic that makes siblings similar. In contrast, parental indirect genetic effects give a more precise idea of effects that originate in the parents rather than general social factors (Cheesman, Eilertsen, et al., 2020). A positive difference between the two estimates therefore suggests a role for other environmental factors, such as school, neighbourhood, and wider social background effects. However, comparison is complicated because parental indirect genetic effects only capture the part of the parental effect that is correlated with parental SNPs or polygenic scores and may also be biased by assortative mating and population stratification.

In future, it would be informative to compare estimates of parental indirect genetic effects to estimates of the familial environment component (F) from extended twin family designs (ETFDs) (Coventry & Keller, 2005; Keller et al., 2010). In comparison to the shared environment estimate (C) from twin studies, F is a much closer analogue to the indirect genetic effect estimated using genomic approaches because it estimates parental effects while controlling for shared twin/sibling environments. If parental indirect genetic effects estimated with Trio‐GCTA were compared to F estimated with ETFDs in the same data, this could quantify the ‘missing heritability’ of parental effects i.e., the amount of variation in the environment due to parental genetics that cannot be captured by SNPs (albeit with the caveat that assortative mating is controlled for in the former but not the latter model).

Triangulation, supported by data simulation, has revealed how sibling, trio and adoption designs for estimating indirect genetic effects (discussed in the next section), although previously assumed to have the same properties, are differentially biased (Demange et al., 2022). Adoption estimates are less prone to population stratification and assortative mating. Where adoption estimates are lower than those based on the other designs, this suggests that these two biases are at play, potentially in combination with prenatal indirect genetic effects (which are not captured in the adoption design).

## INTEGRATING FAMILY AND GENOMIC DESIGNS

While triangulation between classic family‐based methods and population‐based genomics is valuable, it is counterproductive to maintain a divide between these approaches. Integrating family data and cutting‐edge genomic tools allows challenges to be overcome, and knowledge to be gained that was not achievable with one set of designs alone.

### Integrating designs to address missing heritability

Recent whole‐genome sequence data suggests that, at least for height and BMI, missing heritability can be accounted for by rare genetic variants (Wainschtein et al., 2022). Effects of rare variants can also be detected through genomic analysis of relatives. Whilst population‐based approaches cannot detect effects of variants that are uncorrelated with common genotyped SNPs, the GREML‐KIN method uses close genetic relatives to increase the correlation between genotyped SNPs and causal variants (Hill et al., 2018). GREML‐KIN analyses, which thus capture effects of additional non‐genotyped genetic variation such as rare and structural variants, have replicated twin‐study heritabilities for neuroticism and educational attainment in two cohorts (Cheesman, Coleman, et al., 2020; Hill et al., 2018). This hybrid genomic‐family method is more useful than conventional twin or SNP heritability estimates because it reveals how much heritability is due to common versus rare genetic variation, and therefore indicates where in the allele frequency spectrum to look for genetic associations. However, confounding due to indirect genetic effects, assortative mating, and population stratification cannot be ruled out. Additional methods with different/fewer biases are needed to infer the role of rare variation.

A new potential explanation for low SNP heritability of psychopathology has arisen from family‐based genomic‐relatedness methods for estimating direct and indirect genetic effects. Importantly, methods such as Trio‐GCTA (Eilertsen et al., 2020) allow estimation of covariance between direct and indirect genetic effects. This parameter measures how much genes shared by parents and children act in the same or different directions depending on who is carrying those genes. This covariance (i.e., gene‐environment covariance) can be negative. For example, genetic variation that increases risk for conduct problems through the child has an opposing environmentally mediated effect reducing conduct problems through the parent (Eilertsen et al., 2022). Conventional population‐based SNP heritability is underestimated when covariance is negative because it is the sum of the child genetic effect, half the parental indirect genetic effect, and the covariance between offspring and parental effects. For example, when parental SNP data are not modelled, the estimated single‐component SNP heritability of child depression is suppressed by half (0.10 rather than 0.20) (Cheesman, Eilertsen, et al., 2020). Empirical and theoretical research is required to quantify how much this explains missing heritability for various traits, and to interpret the implications of negative covariance for family dynamics, socialisation, and evolutionary biology. Importantly, it is family‐based genomic data that make this possible, because estimating the covariance in pedigree data involves modelling phenotypic information on both parents and children which could be vulnerable to generational differences in genetic effects.

### Integrating designs to understand genetic, environmental, and confounding effects

Genomic data on closely related family members provides valuable information for disentangling genetic, environmental, and confounding effects. For example, siblings can inherit both, one or none of the same parental alleles at each locus, which means that siblings can share between ∼38% to ∼62% of their genome rather than exactly 50% (Visscher et al., 2006). Genetic differences between siblings, which occur at (quasi‐) random because of mendelian segregation, are a kind of ‘within‐family’ genetic variance. These differences, not measurable before the genomic era, have been used to estimate direct genetic effects of PGS and individual SNPs (Howe et al., 2022) on psychopathology. This within‐family genetic influence is not expected to be related to between‐family confounding effects of population stratification, indirect genetic effects, and assortative mating. When within‐family estimates are contrasted with population‐level estimates, the combined magnitude of these three factors can be determined. Parent‐offspring/trio designs (Eaves et al., 2014; Eilertsen et al., 2020; Young et al., 2018) and genomic adoption designs (Cheesman, Hunjan, et al., 2020) are similarly valuable for disentangling genetic and environmental explanations. Notably, the added value of genomic data in the context of classic family structures is great. For example, we could now measure adopted children's genetic risk for psychopathology directly using PGS, rather than indirectly proxying this through biological parent phenotypes. It is important to consider similarities and differences in how these three methods (sibling, trio, and adoption) estimate direct genetic effects, indirect genetic effects, and confounding effects. These methods have been described and compared previously—see the Triangulation section and (Demange et al., 2022).

The significance of the combined family‐genomic methods described above does not stop at estimating direct genetic effects. Just like the trajectory of twin studies from simple univariate decompositions to more sophisticated questions, we are moving beyond simply quantifying direct genetic effects, towards using within‐family genetic variation as a principled tool for understanding developmental gene‐environment interplay. A prime example is gene‐environment interaction, notoriously difficult to study, partly due to concerns about gene‐environment correlation (even with twin data). When the effect of a within‐family polygenic score for ADHD is shown to be contingent upon the school environment (Cheesman et al., 2022), this provides strong evidence for gene‐environment interaction because genetic risk for ADHD is effectively randomised across schools. Indeed, the interaction cannot be explained by parental selection of school environments (an example of active gene‐environment correlation). It is impossible to perform such an analysis using twins because most twins attend the same school. As such, family‐based genomic research lets us ask questions that have been unanswerable with only family‐based or only genomic methods.

## DISCUSSION

Family‐based studies are the foundation and justification for genetic research on developmental psychopathology. Although the development of large‐scale projects aimed at identifying specific genetic risk factors led the continuing utility of twin and family data to be questioned, there is now a growing awareness that knowledge and data from such studies are still extremely valuable (Friedman et al., 2021; Young et al., 2019). With a view to encouraging family‐based genomic research on developmental psychopathology, we have delineated three ways forward. Specifically, we have pinpointed how, by using existing knowledge from family studies, triangulating with family studies, and integrating with family studies, genomic research can become better equipped to recover missing heritability, and to understand the gene‐environment interdependencies shaping psychopathology.

Integrated genomic data on families is essential for distinguishing direct genetic mechanisms from alternative processes including parental indirect genetic effects. As such, a key long‐term goal is the development of large multi cohort family‐based samples with which to perform within‐family GWAS for developmental psychopathology, ideally representing diverse ancestries. Such research will provide knowledge of SNP associations that could be used for many kinds of downstream analyses including PGS and mendelian randomisation.

Beyond GWAS, there is much progress to be made in addressing more subtle questions about psychopathology using genomics. Here, developmental psychologists and family‐based researchers are best placed to contribute. For example, rich knowledge of socioemotional development provides inspiration on testable mechanisms explaining parental indirect genetic effects. This could yield convincing causal evidence relevant to intervention and treatment of childhood psychopathology. Indeed, family‐based genomic studies are starting to incorporate environmental measures, just as twin studies originally brought environmental measures into genetics (Plomin & Viding, 2022). Family‐based quantitative genetics also charts the direction for genomic research on developmental psychopathology with respect to more comprehensive modelling of family relationships. For example, a genomic method based on avuncular correlations (e.g., child and her aunt) like the children‐of‐twins design (Nivard & Lyngstad, 2022) can estimate parental indirect genetic effects on psychopathology over and above population stratification and assortative mating.

However, there are barriers to moving family‐based strategies from gold‐standard to mainstream. Since only around half of the genetic variation in a population is within‐families, larger samples of families are required to obtain the same study power as standard population‐wide genomic analyses. Sample sizes of genotyped relatives are currently low because families are harder to recruit than unrelated individuals. Luckily, the rise of national biobanks means that family members often happen to be included.

In sum, we have defined three key fronts of the growing movement in genomics towards incorporating family‐based research: using knowledge, triangulating, and integrating. We have shown how, in these three ways, family information can advance the genomics of psychopathological phenotypes, where issues of missing heritability and disentangling gene‐environment interplay are particularly pressing. Genomic approaches give us detailed information on individual‐level genetic risk and protective factors and come with a powerful large‐scale collaborative research culture. Family‐based approaches capture total latent genetic and environmental influences, provide more principled ways to understand genetic and environmental influences, and stem from a literature emphasising the important roles of gene‐environment interplay and development. Now we have the best of both worlds.

## AUTHOR CONTRIBUTIONS

**Rosa Cheesman**: Conceptualization; Writing – original draft. All authors provided comments and approved the final manuscript.

## CONFLICTS OF INTEREST

Eivind Ystrom is a Joint Editor for JCPP Advances. The remaining authors have declared that they have no competing or potential conflicts of interest.

## ETHICAL CONSIDERATIONS

Not applicable, since no human participants were involved in this review article.

## Data Availability

Not applicable, since no data were collected or analysed for this review article.
